# Supplementary figures and images for: Comparative analysis of fatty acid metabolism based on transcriptome sequencing of wild and cultivated Ophiocordyceps sinensis
Source: PeerJ. 2021 Jul 1;9:e11681. doi: 10.7717/peerj.11681 (PMC8255070; doi:10.7717/peerj.11681)

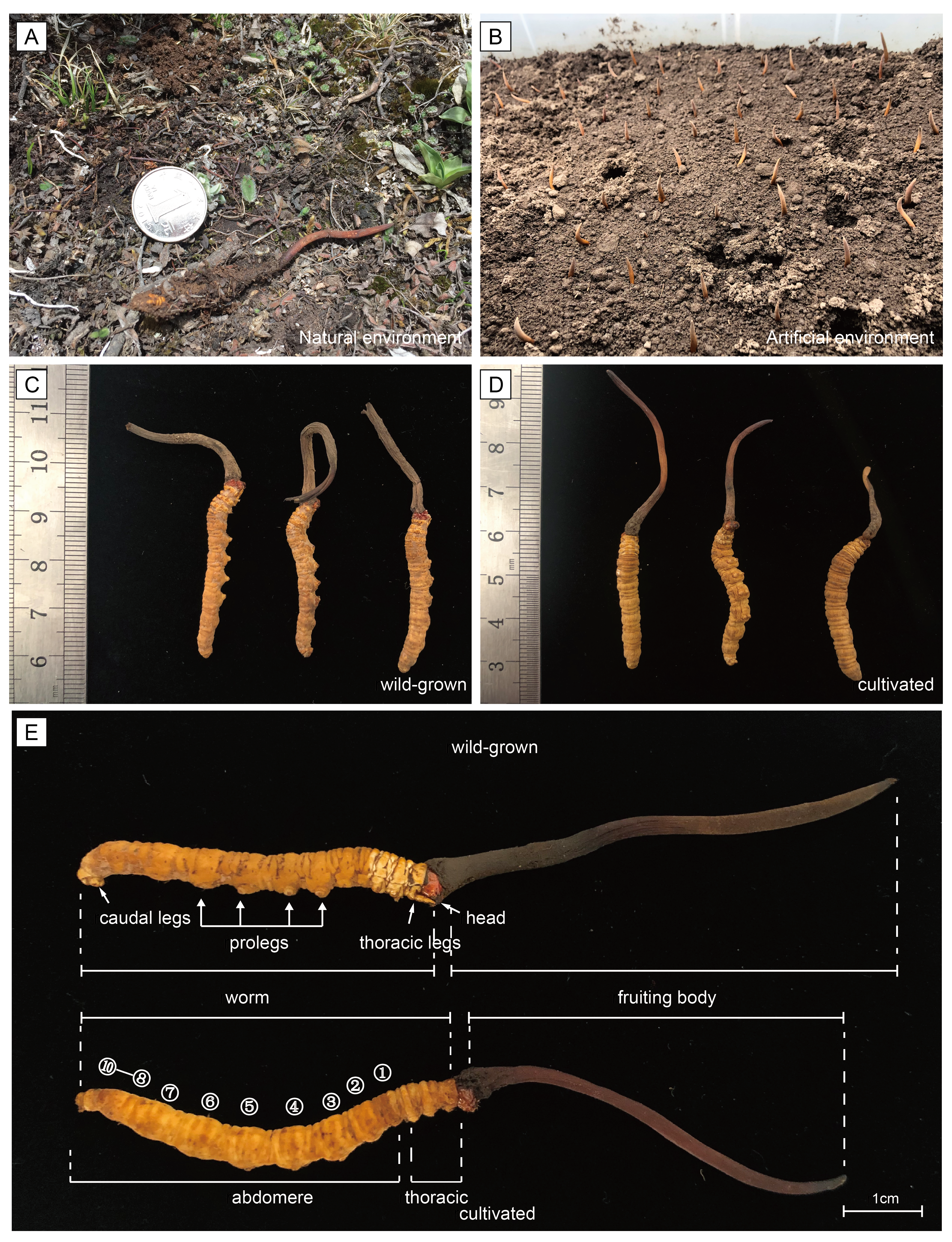

Supplement: Supplemental Information 1 [file peerj-09-11681-s001.png]

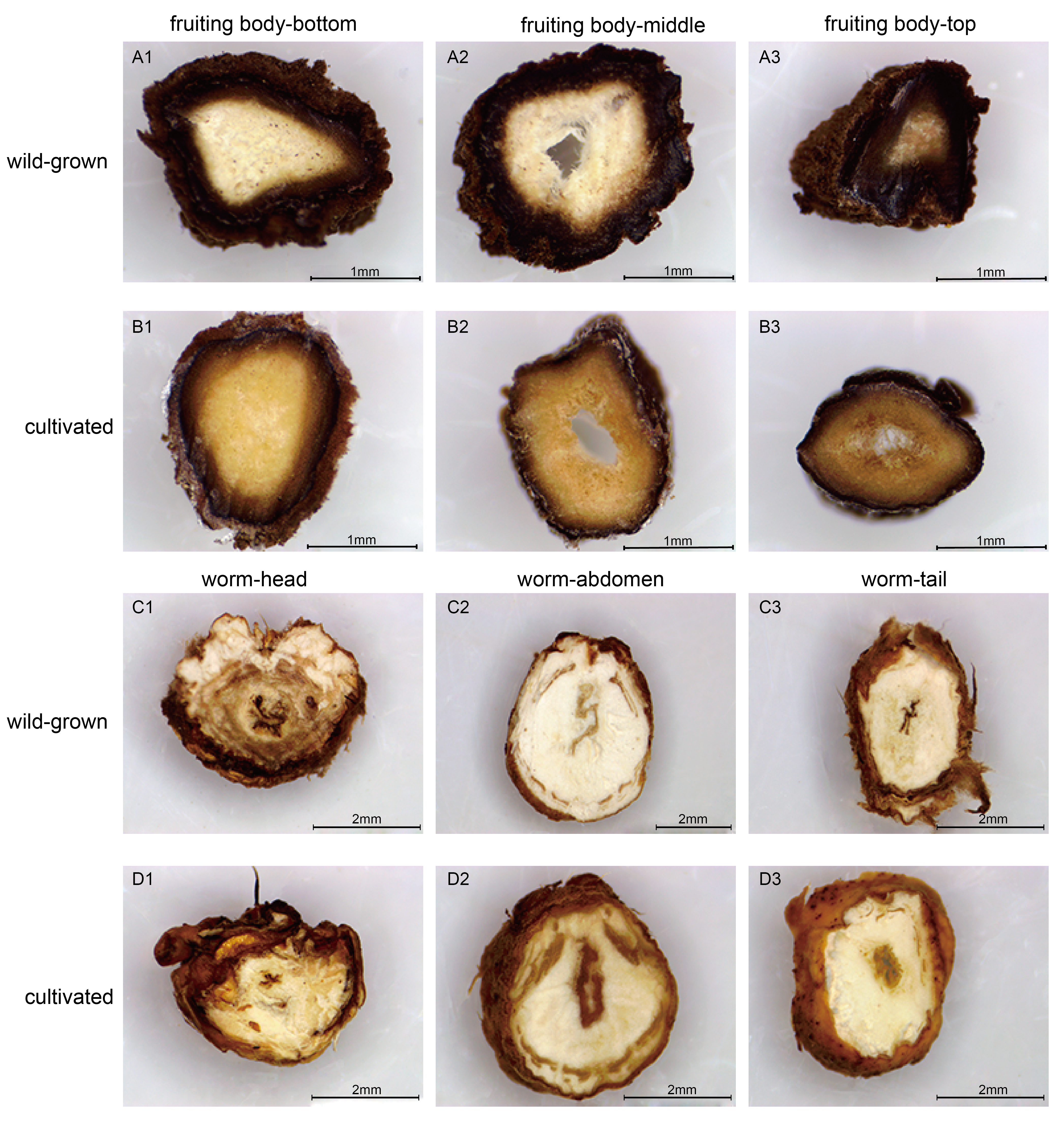

Supplement: Supplemental Information 2 [file peerj-09-11681-s002.png]

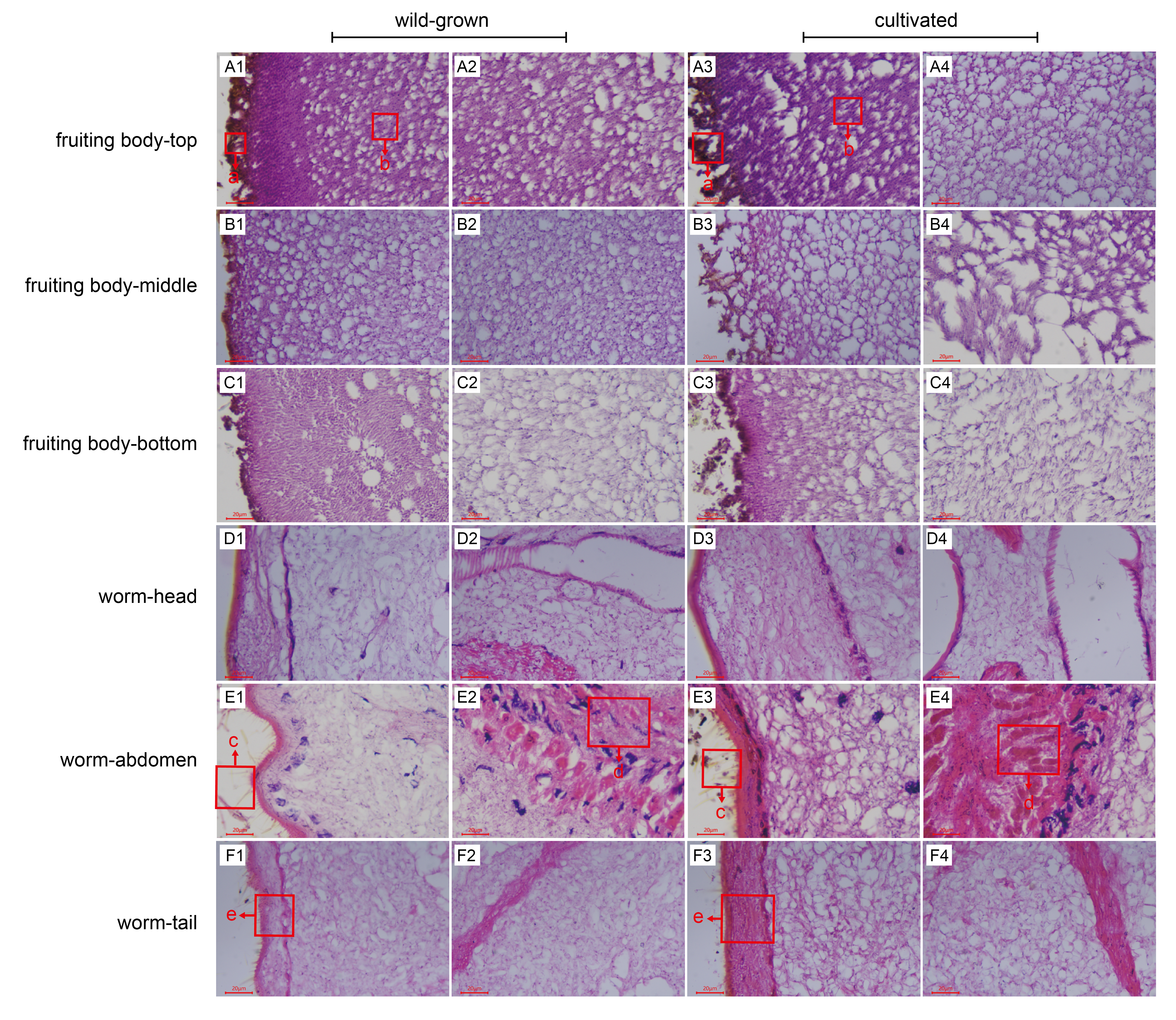

Supplement: Supplemental Information 3 — a, the hyphae germinated on the outer wall of the fruiting body; b, the cross-section of the hyphae inside the fruiting body; c, the fine bristles on the outer wall of the worm; d, the digestive tract in the body cavity of the worm; e, the structure of the outer wall of the worm. [file peerj-09-11681-s003.png]
